# Photon-counting CT for radiotherapy: Qualitative assessment of potential clinical value by semi-structured expert interviews

**DOI:** 10.1016/j.ctro.2026.101135

**Published:** 2026-02-23

**Authors:** Thiele Kroes-Kobus, Linda Rossi, Joost J.M.E. Nuyttens, Dirk K.M. de Ruysscher, Arlette E. Odink, Manouk J.J. Olofsen-van Acht, Ilse M.N. de Pree, Edwin H.G. Oei, Joris B.W. Elbers, Michiel Kroesen, Anke W. van der Eerden, Jan Willem M. Mens, Steven H.J. Nagtegaal, Dennis de Witte, Martine Franckena, Sophie F.N. Vermaas – Fricot, Judith G. Middelburg - van Rijn, Alejandra M. Mendez Romero, Francois E.J.A. Willemssen, Ivo G. Schoots, Patrick Wohlfahrt, Marcel van Straten, Mischa S. Hoogeman

**Affiliations:** a Department of Radiotherapy, Erasmus MC Cancer Institute, University Medical Center Rotterdam, Rotterdam, the Netherlands; b Department of Radiology & Nuclear Medicine, Erasmus MC, University Medical Center Rotterdam, Rotterdam, the Netherlands; c Department of Research and Development, Holland Proton Therapy Centre Delft, Delft, the Netherlands; d Siemens Healthineers, Varian, Cancer Therapy Imaging, Forchheim, Germany

## Abstract

**Background and purpose:**

Photon-counting CT (PCCT) has several potential benefits that can considerably improve the radiotherapy workflow, like availability of intrinsic spectral data and high resolution imaging. It is unknown which treatment sites in radiotherapy will benefit the most from PCCT. This study aimed to identify clinical applications and treatment sites where PCCT may have the most clinical value.

**Materials and methods:**

Semi-structured interviews with expert teams were conducted about eight treatment sites. Each expert team consisted of 2 radiation oncologists and 1 radiologist with expertise in specific treatment site. During each session, PCCT images were shown to demonstrate specific PCCT features: instantly changing the energy from 40 to 190 keV with a slider, iodine maps, contrast-agent removal and high-resolution images. Next, the experts scored ten statements about clinical applications for added clinical value on a 5-point Likert scale, from 0=No added clinical value to 4 = Indispensable for the clinic.

**Results:**

A total of 80 scores were collected about ten statements and eight treatment sites. The statements about the use of spectral data scored highest for added clinical value, while reducing imaging dose scored lowest. The radiotherapy treatment sites with the highest scores were bone, head and neck and lung tumors, while lowest was prostate where no added value was found.

**Conclusion:**

Semi-structured interviews identified advantages of PCCT that may have the most added clinical value for radiotherapy: the use of spectral data and increased resolution. Head and neck, bone and lung tumors are the clinical applications which were scored highest in terms of added clinical value and these could therefore be the first areas for clinical implementation.

## Highlights

• Semi-structured interviews provided insight in the potential added value of PCCT for radiotherapy.
• Head and neck, bone and lung tumors had the highest potential of having added clinical value.
• Availability of spectral data and high-resolution imaging contributed most to the added clinical value.

## Introduction

Computed tomography (CT) is the primary imaging modality in radiotherapy, due to a short scan time, good spatial resolution and good relation between CT numbers in Hounsfield units (HU) and relative electron density [1]. However, the soft-tissue contrast on CT is limited, which led to a search for better imaging. This resulted in the introduction of magnetic resonance imaging (MRI) in radiotherapy and its application is still expanding [2]. MRI brings other challenges, like longer scan times, system availability and costs. Therefore, advances in the CT simulation procedure are of special interest. If this could eliminate the need for additional planning imaging, this would not only save time and costs, but also minimizes uncertainties due to discrepancies in image fusion. A recent advancement in CT is the introduction of photon-counting detectors (PCD), which made photon-counting CT (PCCT) possible and opened a range of applications based on the advantages over energy-integrating detectors [3], [4].

In a PCD, each incoming photon is registered by converting it directly into an electric signal of which the height depends on the energy of the photon [5]. This way, the energy-dependent attenuation can be derived and used to create virtual mono-energetic images (VMIs) at a specific energy in the range of 40 to 190 keV. Typical examples of potential benefits are the enhancement of iodine to increase soft-tissue contrast for low-energy VMIs (<70 keV), due to the relatively large attenuation of iodine at low energies, and the reduction of the beam hardening artefact at higher energies. Spectral data can also be used for material decomposition, for example to extract the iodine signal from contrast-enhanced images, resulting in an iodine map and a virtual non-contrast (VNC) image [6].

Another advantage of a PCD is its relatively small detector elements, allowing for ultra-high-resolution imaging (highest resolution: 0.11 mm in-plane) [7]. Furthermore, the registration of photons above a specific energy threshold by a PCD enables the separation between signals from individual photons and electronic noise. This allows for a higher signal-to-noise ratio (SNR), which could be exploited to improve image quality in relatively low signal situations (e.g. low dose or high attenuation).

These advantages of PCCT are altering the field of radiology and could also impact radiotherapy. CT plays a crucial role in the radiotherapy workflow as it is used for contouring, treatment planning, the reference image for image-guidance and follow-up. Therefore, a change in CT reconstruction affects the entire radiotherapy chain [8]. Implementation of a new technique in radiotherapy takes significant resources from implementation teams and researchers. Moreover, the total cost of ownership of PCCT systems is higher than for a conventional CT. It should therefore not be pursued for novelty, but for clinical benefit. Demonstrating the clinical benefit in proof-of-principle trials is laborious, costly and time-consuming and requires purchasing a system. Therefore, it is essential to first determine whether and where clinical benefits are to be expected. In this study, the potential benefit of PCCT for radiotherapy is assessed by conducting semi-structured interviews with clinical expert teams. The aim is to identify clinical applications and tumor sites where PCCT may have the largest added clinical value. The outcome can be used to select the best tumor sites for further investigation and clinical introduction.

## Methods

### Patient inclusion

At the Department of Radiology & Nuclear Medicine of Erasmus MC, two PCCT systems (NAEOTOM Alpha, Siemens Healthineers, Erlangen, Germany) have been in operation since 2021 and 2024. For this study, diagnostic PCCT images of radiotherapy patients were reviewed. Patients that fulfilled the following criteria were considered: 1) patient received a scan on a PCCT scanner in the period between 07-04-2021 and 16-05-2024, 2) patient started radiotherapy within 7 months after the PCCT scan, 3) the volume treated with radiotherapy was within the PCCT scan, 4) a spectral post-processing dataset was available, and 5) patient gave informed consent for the use of clinical data for research purposes. A maximum of seven months between the acquisition of the PCCT and onset of radiotherapy was set to facilitate inclusion of patient with a visible tumor. The local ethics committee approved this study (Institutional review board protocol METC 2021–0847).

In our institution, radiation oncologists are specialized in one or more radiotherapy focus areas: breast, urology, palliation, gastro-enterology, lungs, gynecology, head and neck, sarcoma, neurology, hematology and skin. Patients who fulfilled the inclusion criteria were sorted by focus area and focus areas with at least 4 patients were included in the study. Some focus areas involve various organs in which the target volume can be located (e.g. bladder or prostate for urology) and each organ can have its own imaging-related needs and challenges. To focus the discussion for such cases, the clinical value was assessed for the largest subset of patients or the subset with most CT-related imaging challenges. These were patients with bone metastasis for palliation, cervical cancer patients for gynecology, liver cancer patients for gastro-enterology and for urology, two targets were considered: prostate and bladder cancer.

### PCCT images

The image data of 31 patients were used. As diagnostic PCCT-images were reviewed, the image acquisition and reconstruction parameters were optimized for specific clinical indications (e.g. diagnosis, staging, follow-up imaging). For 28 of 31 patients an iodine contrast agent was used. Ultra-high-resolution reconstructions were mainly part of the diagnostic protocol of specific bone examinations, but also for other indications a high matrix (e.g. 768x768 or 1024x1024) and sharp kernel (e.g. Br68) was used diagnostically. One or more patients with a large matrix and sharp kernel were included with bone, lung, head and neck and breast tumors. For diagnostic purposes, by default for all patients a spectral post-processing dataset was saved. This is a data format that stores the spectral information and enables the use of these data at a later stage.

### Semi-structured interviews

Ten statements were drafted about potential clinical applications of PCCT for radiotherapy, reported in Table 1. A 5-point Likert scale for added clinical value compared to a conventional CT was used for scoring the statements: 0=No added clinical value, 1=Some added clinical value, 2 = Added clinical value, 3=Substantial clinical value, and 4 = Indispensable for the clinic, or ‘I don’t know / not applicable’, when preferred. Part of the statements involve features that could be reviewed on the PCCT-images, while also statements were included for which no visual review possible was, as the potential is unknown (e.g. toxicity prediction) or there are no clinical images yet to show (e.g. spectral 4DCT). For the latter, the experts score based on their experience with conventional CT expanded by the visual review of the available PCCT-images.

**Table 1:** The 10 statements of clinical applications for which the clinical value was evaluated. The statements involve aspects that were shown, like iodine maps, but also applications for which the clinical value is hypothetical, as the potential is unknown (e.g. toxicity prediction) or there are no clinical images yet to show (e.g. spectral 4DCT).

	Statements
A	Spectral information has clinical value in routine CT simulation
B	The creation of virtual mono-energetic images has clinical value for contouring
C	The removal of contrast media in contrast-enhanced CT has clinical value
D	Contrast media images (iodine map) has clinical value (e.g. separation tumor from bone enhancement)
E	Images acquired with increased resolution has clinical value
F	Saving imaging dose has clinical value
G	The acquisition of spectral respiratory 4D has clinical value
H	Dose optimization incorporating organ physiology (lung ventilation and perfusion) for smart sparing of healthy tissue has clinical value
I	Prediction of RT response and toxicity on the basis of PCCT images has clinical value
J	Dose painting based on PCCT images has clinical valuable

For each focus area, an expert team was formed consisting of two radiation oncologists and one radiologist. The physicians were all experts in the specified tumor site and not necessarily experts on PCCT or spectral imaging. However, all radiologists are accustomed to reading images acquires on the PCCT. One resident in medical physics led all discussions and showed the PCCT images. The duration of each session was between 60 and 90 min.

The semi-structured interview consisted of five steps:

1. The experts shortly discussed the currently used imaging modalities for contouring of the tumor site and the difficulties faced in this task.
2. The resident exposed the experts to the statements to make the team aware of the aspects that will be evaluated.
3. The resident showed PCCT images of 4–6 patients in syngo.via (Siemens Healtineers, Erlanger, Germany) to give an impression about the possibilities of PCCT. In syngo.via the spectral post-processing data can be used to instantly change the energy of the reconstruction from 40 to 190 keV with a slider. The resident started with a reconstruction energy of 70 keV, which is similar in appearance as a planning CT at 120 kV. As a direct comparison with the planning CT was not part of the assessment, scrolling through the 70 keV VMI of the patient gave an impression of the contrast in a planning CT. Then the reconstruction energy was changed between 40 to 190 keV. Next, the iodine map, VNC, overlay of the iodine map on the VNC, and high-resolution images (when available) were shown. Experts were given the opportunity to go scroll through the patients as well.
4. The experts discussed about the statements and reached consensus on the scoring of added clinical value on the 5-point scale. The resident noted remarks and comments of the discussion and made a summary.
5. The resident shared the consensus scoring and summary of the discussion with the expert team after the meeting to allow for adaptation in case needed.

The scores were summed per statement and per tumor site. If the statement was scored ‘I don’t know’, it was not included in the sum.

## Results

Semi-structured expert team interviews were conducted about tumors in the breast, prostate, bladder, bone, liver, lung, cervix and head and neck region. The tumor sites sarcoma, neurology, hematology, and skin were excluded as not enough patients fulfilled all criteria. For the lung and urology expert team interview, not all experts could be present at the same time. In these cases, two sessions were held. During the first session with urology experts, the statements were scored and this was assessed in the second session, which did not lead to alterations. For the lung experts, two separate scorings were performed. These scores were averaged; except when the answer during one session was ‘I don’t know’, then the score of the other session was used.

The opinion of 20 experts was collected (7 radiologists and 13 radiation oncologists). Ten scores for eight treatment sites were collected resulting in a total of 80 scores. Of these, 27 received a score of 1 or higher, indicating at least some added clinical value.

**Fig. 1:**
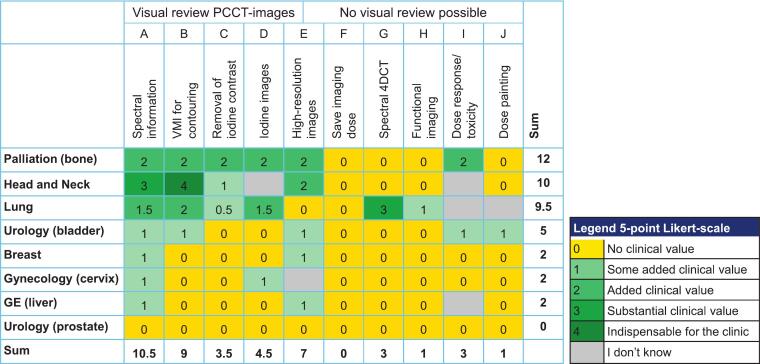
Overview per radiotherapy target of the added clinical value for the different PCCT applications.

The tumor sites with the highest summed score were bone, lung and head and neck, while no added value was scored for the prostate (Fig. 1). The applications with the highest summed score were the use of spectral information, VMI for contouring and high-resolution images.

### Spectral data

**Fig. 2:**
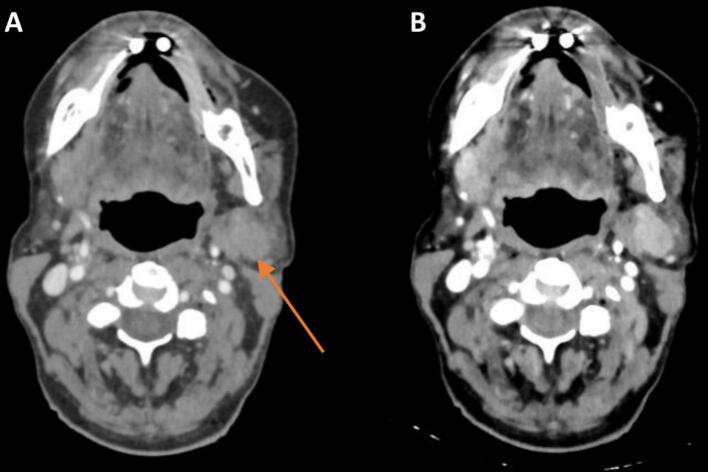
Example of the enhanced iodine contrast at low keV virtual mono-energetic images. *A) Virtual mono-energetic image at 70 keV, which is equivalent to a conventional CT image acquired at 120 kV (typically used for RT planning). Arrow indicates the lesion. B) Virtual mono-energetic image at 40 keV with improved iodine contrast in the tumor, which may lead to a more reliable tumor delineation, in this case especially at the medial border. Both images are displayed at window width 400 and window level 40.*

**Fig. 3:**
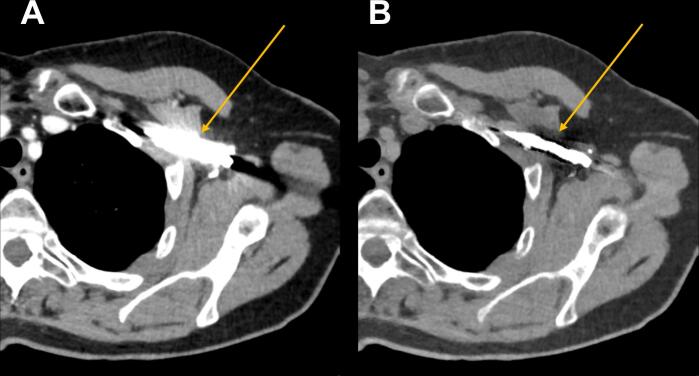
Example of the reduction of the artefact due to the high iodine concentration in the axillary and subclavian veins (arrow). A) Virtual mono-energetic image at 70 keV equivalent to a conventional CT image acquired at 120 kV. B) Virtual mono-energetic image at 190 keV leading to a smaller artefact, which could improve visualization of the adjacent lymphatic tissue. Both images are displayed at window width 350 and window level 40.

The use of spectral information for CT simulation was scored for all target sites between *some* and *substantial added clinical value*, with the exception of the prostate. Lung, bone, and head and neck scored highest (Fig. 1). This statement encompassed all uses of spectral data: VMIs, VNCs, but also the use of a slider to instantly change the energy and optimize the contrast for the individual patient. The use of spectral data was further specified in statements B-D (Table 1), of which the creation of VMIs for contouring had the highest sum (Fig. 1). The experts appreciated the improved iodine contrast at low keV VMIs for better definition of the extend of the tumor (example in Fig. 2). Six of the 31 patients had beam hardening artefacts in their images due to dental or prosthetic implants or markers. The experts that viewed these images had the opinion that dedicated metal artifact reduction (MAR) reconstructions handled these artifacts better than high keV images (without MAR). Some experts identified a small benefit in high keV reconstructions to reduce the artefact caused by the high iodine concentration after contrast administration in the axillary and subclavian veins (example in Fig. 3).

**Fig. 4:**
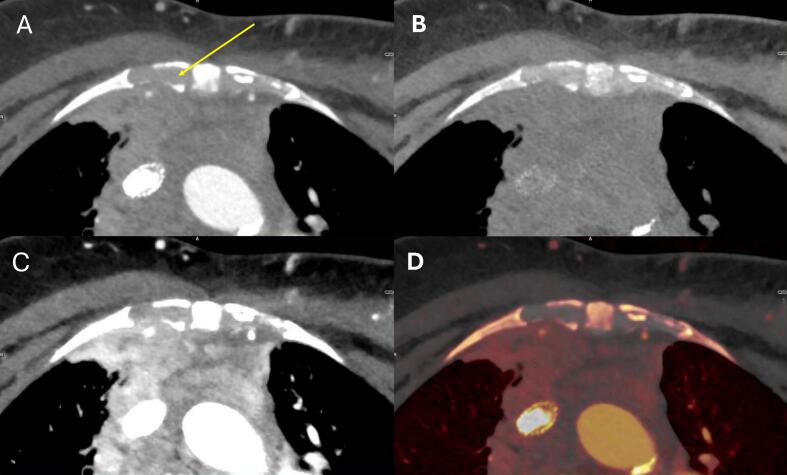
Example of a mediastinal tumor where spectral information might be used to rule out cartilage involvement (arrow). Scan acquired with iodine contrast administration. A) Virtual mono-energetic image at 70 keV equivalent to a conventional CT image acquired at 120 kV (typically used for RT planning). There is asymmetry of the cartilage which could either be normal anatomy or tumor involvement. B) Virtual non-contrast image, where the cartilage is hyperdense to the tumor C) Virtual mono-energetic image at 40 keV improves separation between tumor and the non-enhancing cartilage. D) Color overlay of the iodine map over the virtual non-contrast image, showing the difference between enhancing and non-enhancing tissue. A and C are displayed at window width (WW) 500 and window level (WL) 80. The virtual non-contrast in B and D is displayed as WW 400 and WL 40 with an overlay of the iodine map in D) at WW 500 and WL 250.

The lung experts saw potential in the use of the iodine map for improved visualization of mediastinal tumors (example Fig. 4). The head and neck experts deemed the VNC maps helpful for specific cases to differentiate between vessels and tumor. Also, a practical use of VNCs was mentioned: improve the workflow by eliminating the need to delineate and override the density on strongly enhancing organs or save time by directly transferring contours from a contrast to a virtual non-contrast scan without adaptation.

### High-resolution imaging

Of the non-spectral applications, high-resolution images had the highest summed score for added clinical value. Bone and head and neck tumors were the tumor sites with the highest score (Fig. 1). A higher through-plane resolution, i.e., thinner slices, was not of interest, as this would mean more contouring, without an expected improvement in target delineation.

### Dose saving

The imaging dose potential scored *no added clinical value* by all experts (Fig. 1), but several experts deemed reducing imaging dose in follow-up (PC)CT scans relevant.

### Respiratory 4DCT

The use of spectral respiratory 4DCT scored *substantial added clinical value* for the lung, but *no added value* by all other expert teams, as either no 4DCT scan is used or the spectral information is not of interest (Fig. 1)*.* The lung experts see value to have a good quality contrast-enhanced and non-contrast scan in the same phase, but there was doubt on the feasibility of the post-processing given the change in contrast concentration over the long duration of the 4DCT scan*.*

### Functional imaging

The use of PCCT to determine which part of the tissue (e.g. of the lungs or liver) was most functional was discussed. This technique could be used to spare this part of the organ by steering the dose through less functional tissue. This was not scored to have added clinical value by any of the expert teams except for the lung (Fig. 1).

### Response and toxicity prediction

Only the bone and bladder expert teams scored at least *some added clinical value* for response or toxicity prediction*.* Most expert teams doubted that PCCT could bring more than conventional CT.

### Dose painting in the target

Only the lung expert team did foresee *some added clinical value* for PCCT for dose painting, the others experts did not expect PCCT would have added value (Fig. 1).

## Discussion

In this study, semi-structured expert team interviews were conducted to identify the applications and radiotherapy treatment sites where PCCT might have most clinical value in radiotherapy. Introducing a new technique in radiation oncology is laborious, time-consuming and costly. Therefore, identification of the most promising applications and targets is essential.

The PCCT applications that scored highest for added clinical value were the usages of spectral data. Of these, VMIs scored higher than the use of iodine maps and VNCs. The VNCs and iodine maps need to be used with care due to limitations in material decomposition assigning a part of the bone attenuation to iodine. However, this is manageable, as it has been demonstrated that VNC images result in comparable dose distributions as contrast-enhanced CT-images and lead to improved CBCT registration [9]. The combination of low keV VMIs with a contrast agent enhances tumor visibility [10]. This was indicated as potential method to improve target delineation and reduce healthy tissue irradiation.

The target areas that scored the highest clinical value were head and neck, bone and lung tumors. Least added value was expected for prostate, liver, cervical and breast tumors. For breast tumors, assessment of the value was complicated by the fact that the available breast PCCT scans were pre-operative scans, while breast experts usually deal with post-operative CT scans. For the prostate, liver and cervix, MRI plays an important role in diagnostics and target delineation. In general, the soft-tissue contrast of MRI was preferred over the spectral PCCT data for these tumors. For head and neck cancers, MRI also plays an important role; however, the experts appreciated the improved tumor enhancement at low keV VMIs compared to the typical 120 kV scan. This was assessed as *indispensable* for the clinic. This makes head and neck tumors an interesting target for further research on the complementary or overlapping value of MRI and PCCT. Both the lung and bladder experts also appreciated the ability to change the enhancement of the iodine contrast for the individual patient. As the brightness of the contrast varies per patient due to differences in physiology, they valued the option to change this interactively. For the bone, there was also interest in the developments on PCCT for quantification of bone marrow edema as this could improve tumor delineation. Accurate tumor delineation is also of importance in a palliative setting. Bladder cancers may also be interesting for further analysis with PCCT as CT plays an important role in diagnostics and staging of the disease and scored intermediate on added clinical value.

Spectral data can also be acquired using DECT [11], [12], but its use in radiotherapy is still limited [13]. However, there are differences between PCCT and DECT and their value for radiotherapy may not be the same. On most DECT systems, the spectral data is acquired in two scans with a low and high tube voltage. The acquisition of these two scans is implemented by vendors in different ways, each with its own strengths and weaknesses [14]. One weakness of most DECT approaches is the sensitivity to motion, as a high temporal resolution is needed between the two acquisitions for adequate spectral processing. For moving anatomies, the intrinsic spectral data gives PCCT an advantage over most DECT systems. This advantage is smaller for targets that move very limited, like for head and neck cancer patients positioned in a mask, although swallowing and breathing can still cause motion artifacts. Phantom studies have demonstrated improved low-contrast detectability in abdominal protocols and less dependence of CT numbers (HU) on object size for PCCT over DECT [15], [16]. If the impact of PCCT on radiotherapy will be larger than DECT, remains to be seen, but its big impact on the field of radiology cannot be ignored.

To fully benefit from spectral data, multiple image reconstructions optimized for different aspects of the RT workflow might be required [17]. These could be a low-energy image for delineation and an electron-density map for dose calculation. The data handling of different datasets might pose a challenge, which asks for novel solutions [13].

In this study, semi-structured expert team interviews were conducted to identify areas for further investigation and clinical introduction of PCCT in radiotherapy. This approach prioritized qualitative information over quantitative measures. Others have also used interviews to obtain expert opinions [18], [19], [20], although these interviews were aimed at evaluating a current procedure. Another approach is the Delphi method (e.g. [21]), where key opinion leaders are interviewed over several rounds. The latter might not be the most appropriate for identifying the most promising areas of implementation on a departmental level, where the experience with the new technique is still limited. Interviewing experts is a straightforward implementable approach and could be performed before clinical introduction of other new techniques as well. By demonstrating the new possibilities and creating enthusiasm, clinical expert interviews might also help to accelerate clinical adoption of a new technique.

This study had some limitations. This was a single-center study. Although the experts have a vast amount of experience in radiation oncology, the results might not be generally applicable, especially not for institutions that have a different approach for pre-treatment imaging. Furthermore, the number of experts was limited, so personal preferences might have influenced the results. As the PCCT scanner is not in clinical use for radiotherapy, diagnostic PCCT images were not specifically optimized for radiotherapy. Only a limited number of patients per tumor site were viewed, and these might not have been patients that would benefit most from PCCT. Also, not all potential PCCT applications could be demonstrated, like quantification of liver or lung function, which is not performed in our institution. Several studies using DECT have investigated the use of functional imaging for liver or lung aiming at optimal dose distribution to spare the normal tissue [22], [23]. As no pediatric patients are treated with radiotherapy in our institution, we only included adult data. The impact of high-resolution imaging or imaging dose reduction might be significant for pediatric patients. Finally, we did not investigate the potential of PCCT for improved dose calculation or autosegmentation. The impact of more precise electron density for photon therapy or improved stopping-power ratio estimates for proton therapy, has already been evaluated by others in phantom studies [24], [25], [26] and was therefore explicitly not part of this study. This study focused on determining the most promising tumor sites for PCCT and these results can direct future studies into the benefits of quantitative images.

This study revealed that experts value the use of spectral data, especially to create low keV VMIs with a contrast agent to enhance tumor visibility. Patients with lung, bone and head and neck tumors might benefit most from this application. Although additional study into tumor sites that were not evaluated yet is appropriate, these could be the first areas for further quantitative investigations and clinical introduction.

## Declaration of competing interest

The authors declare the following financial interests/personal relationships which may be considered as potential competing interests: Erasmus MC Cancer Institute has a test and feedback agreement with Siemens Healthineers (Erlangen, Germany). Siemens Healthineers had no role in study design, data collection and analysis, and decisions on preparation of the manuscript. Patrick Wohlfahrt is an employee of Siemens Healthineers within the CT research and development section. Outside the scope of this work: The department of radiotherapy, Erasmus MC cancer institute has a research collaboration with Accuray Inc (Sunnyvale, CA, USA), Elekta AB (Stockholm, Sweden), and Varian, a Siemens Healthineers Company (Palo, Alto, CA, USA). MH reports an additional research collaboration with Raysearch, Stockholm, Sweden, and participation in a ThinkTank meeting for Accuracy Inc.
